# Temporal recalibration in schizophrenia: a compensatory timing trap?

**DOI:** 10.1093/nc/niag031

**Published:** 2026-06-22

**Authors:** Ali Aytemur, Ayşen Esen Danacı, Osman İyilikci

**Affiliations:** Faculty of Humanities and Social Sciences, Department of Psychology, Manisa Celal Bayar University, 45140, Yunusemre, Manisa, Turkey; Faculty of Medicine, Department of Psychiatry, Manisa Celal Bayar University, 45030, Yunusemre, Manisa, Turkey; Faculty of Humanities and Social Sciences, Department of Psychology, Manisa Celal Bayar University, 45140, Yunusemre, Manisa, Turkey

**Keywords:** sensorimotor temporal recalibration, delay adaptation, temporal adaptation, schizophrenia, dysconnectivity

## Abstract

Schizophrenia is characterized by widespread neural dysconnectivity and impaired temporal coordination. Despite these pervasive disruptions, patients often maintain coherent perception and sense of agency, particularly during remission. This paradox remains underexplored, as research has mainly focused on symptom emergence rather than the mechanisms supporting preserved conscious experience. We propose a dual-role hypothesis of sensorimotor temporal recalibration, a process of adaptation to temporal asynchronies in sensorimotor integration. Temporal recalibration may serve both as a compensatory mechanism mitigating neural incoherence and, under certain conditions, as a trigger for positive symptoms. We compared clinically stable schizophrenia patients with healthy controls using a visuomotor temporal-order-judgement task following adaptation to sensorimotor asynchronies (0, 150, 300 ms). Patients exhibited significantly greater temporal recalibration than controls. When they experienced asynchrony between their actions and visual outcomes, they adjusted their point of subjective simultaneity more strongly, indicating enhanced adaptation to new temporal relationships. Consequently, after adaptation, patients were more likely to perceive outcomes as occurring before their actions when the adapted asynchrony was transiently reduced, whereas without adaptation they perceived them as following the action. No group differences emerged in just noticeable differences, indicating comparable temporal precision and task difficulty. These findings suggest that patients not only possess an intact recalibration mechanism but may engage it more to counteract neural delays and preserve temporal coherence. Overreliance on this adaptive process, however, may distort the perceived order of actions and outcomes, especially under transient temporal instability in information processing, and contribute to disrupted causality and agency in schizophrenia.

## Introduction

Schizophrenia involves impaired communication (Philips and Silverstein 2003; Friston et al. 2016) and disrupted temporal coordination (Ford and Mathalon 2008; Uhlhaas and Singer 2010) between large-scale brain networks. These disruptions are thought to contribute to the fragmented perception and disrupted sense of agency, the experience of control over voluntary actions and their sensory consequences, in schizophrenia. However, current theories mainly focus on the mechanisms underlying symptom manifestation and often overlook how patients maintain relatively preserved perception and agency when neural timing remains disrupted. Therefore, revealing the mechanisms compensating for temporal coordination abnormalities is essential for understanding perceptual functionality in schizophrenia. In addition, exploring compensatory mechanisms is crucial for identifying factors that may interrupt these processes and contribute to the positive symptoms. In this study, we propose a novel hypothesis that extends current dysconnectivity models by addressing two key issues: how relatively coherent perception is maintained despite temporal incoherence, and how this very compensatory process may contribute to symptom onset under certain conditions.

Temporal coordination is crucial for integrating information across different systems into unified perceptual experience. Studies in healthy participants showed that asynchronous inputs can be integrated into a unified perception by adaptively shifting their timing to overcome temporal discrepancies, a process known as temporal recalibration (Fujisaki et al. 2004; Vroomen et al. 2004; Stetson et al. 2006). Especially, sensorimotor temporal recalibration proposed to be a key mechanism for supporting functional interaction with the environment by compensating temporal discrepancies between motor commands and their sensory consequences (Stetson et al. 2006). In schizophrenia, numerous studies have reported greater levels of structural and functional connectivity abnormalities (Friston et al. 2016) that contribute to temporal asynchrony across neural systems (Uhlhaas and Singer 2010). Notably, these disruptions persist even in clinical remission and shared with patients’ unaffected first-degree relatives or with high-risk individuals (Khadka et al. 2013; Andreou et al. 2014; Wang et al. 2016; Pelletier-Baldelli et al. 2018; Del Fabro et al. 2021; Assaf et al. 2024). In addition, schizophrenia has been associated with demyelination related efference copy delays (Whitford et al. 2012; Pynn and DeSouza 2013; Parlikar et al. 2019) and slowed or less efficient visual information processing (Johnson et al. 2005; Silverstein et al. 2015; Jiang et al. 2021; Adámek et al. 2022; Francisco et al. 2022) which can further contribute to sensorimotor timing asynchronies that threaten the integrity of perception unless actively corrected. These patterns of abnormalities may specifically require increased levels of temporal adjustments for perceptual coherence in the visuo-motor domain since both action initiation and visual processing rely on the integrity of these affected networks. Therefore, investigating whether schizophrenia patients rely on heightened recalibration to maintain perceptual coherence could provide novel insights into the adaptive mechanisms that counterbalance neural dysconnectivity and asynchrony.

While temporal recalibration has been considered as an adaptive process, its potential overuse in schizophrenia may have disruptive consequences. Sensorimotor temporal recalibration studies in healthy participants showed that perceived temporal order of actions and their sensory consequences can be reversed when the adapted temporal asynchrony between them is reduced or removed, leading participants to perceive sensory consequences as occurring prior to their initiating actions (Stetson et al. 2006; Heron et al. 2009; Sugano et al. 2010). Therefore, transient reductions in the adapted temporal asynchronies between motor and sensory components can diminish perceived causality between them and lead to attributing one’s agency to external sources (Timm et al. 2014) as sensory events preceding their initiating actions cannot plausibly be self-generated. In schizophrenia, transient reductions in adapted sensorimotor asynchronies may arise from acute changes in neural processing underpinned by heightened arousal, dopaminergic dysregulation, or transient neural synchrony, that have been linked to psychosis (Uhlhaas et al., 2010; Lodge and Grace 2011; Grace 2012; McCutcheon et al. 2020; Corlett and Fraser 2025). Therefore, this can mimic the findings in healthy individuals and excessive reliance on temporal recalibration mechanism may increase vulnerability to positive symptoms under conditions of transient neural processing change by increasing the risk of temporal order distortions.

We propose a dual role for temporal recalibration in schizophrenia. That is, while overreliance on temporal recalibration mechanism may generally support perceptual stability by compensating increased neural asynchrony, it may also paradoxically increase the risk of temporal order disruptions and potentially contribute to positive symptoms, particularly under conditions of increased neural gain or information speed. This hypothesis bridges dysconnectivity models of schizophrenia with mechanistic accounts of perceptual timing and agency. In the current study, we specifically investigated whether clinically stable schizophrenia patients show heightened sensorimotor temporal recalibration compared to healthy controls, as a potential mechanism for mitigating timing discrepancies between motor actions and their sensory consequences.

## Materials and methods

### Participants

The study included 20 individuals diagnosed with schizophrenia and 20 healthy control participants. Participants with schizophrenia (8 females, 12 males; age: *M* = 38.9, SD = 9.8) were recruited from the outpatient unit of the Manisa Celal Bayar University Hospital Psychiatry Clinic. Diagnosis was established using the Structured Clinical Interview for DSM-IV Axis I Disorders (First 1997), and all patients were receiving outpatient treatment at the time of participation. Inclusion criteria for the schizophrenia group were: (i) no change in psychiatric medication within the past month, (ii) no inpatient psychiatric treatment within the past three months, (iii) no history of electroconvulsive therapy, (iv) no diagnosis of neurological disorders, and (v) no alcohol or substance addiction. Symptom severity was assessed using the Scale for the Assessment of Positive Symptoms (SAPS; Andreasen 1984a; Erkoç et al. 1991a) and the Scale for the Assessment of Negative Symptoms (SANS; Andreasen 1984b; Erkoç et al. 1991b). The control group (8 females, 12 males; age: *M* = 39.6, SD = 9.3) was recruited from the local community and matched to the patient group on age, gender, and years of education. Inclusion criteria for controls included: (i) no history of psychiatric or neurological disorders, (ii) no family history of schizophrenia, schizoaffective disorder, or bipolar disorder, and (iii) no history of alcohol or substance addiction. Four participants from the schizophrenia group were initially excluded due to chance-level performance at the largest stimulus onset asynchronies (SOA) (375 ms) in the baseline condition. Their responses across other SOAs were similarly unmodulated, resulting in near-flat psychometric functions indicative of non-systematic or inattentive responding. These were replaced by newly recruited individuals who met the same inclusion criteria. One of the replacements was also excluded and replaced because of the same reason above, resulting in a final sample of 20 patients with schizophrenia.

Demographic characteristics and clinical data for both groups are summarized in Supplementary Table 1. Medication information was recorded for all patients and is summarized in Supplementary Table 2. Antipsychotic treatment most commonly included aripiprazole (*n* = 9), clozapine (*n* = 9), and paliperidone (*n* = 8), followed by olanzapine (*n* = 4), quetiapine (*n* = 2), zuclopenthixol (*n* = 2), haloperidol (*n* = 1), and risperidone (*n* = 1). Several patients were receiving antipsychotic polypharmacy, with 7 patients on antipsychotic monotherapy, 10 receiving two antipsychotics, and 3 receiving three antipsychotics. Adjunctive psychotropic medications included sertraline, escitalopram, citalopram, lamotrigine, lithium, valproic acid, venlafaxine, amitriptyline, zopiclone, biperiden, and propranolol. Because treatment regimens included both oral and depot/long-acting formulations, detailed dose information is provided in Supplementary Table 2.

The authors assert that all procedures contributing to this work comply with the ethical standards of the relevant national and institutional committees on human experimentation and with the Helsinki Declaration of 1975, as revised in 2013. All procedures involving human subjects/patients were approved by Clinical Research Ethics Committee of the Faculty of Medicine at Manisa Celal Bayar University (Approval No: 429). All participants provided written informed consent prior to participation.

### Materials and experimental procedure

For the experimental task, participants sat in front of a computer screen (144 Hz, 27 inch). The temporal recalibration task was presented using OpenSesame 4.0 (Mathôt et al. 2012) on a high-performance gaming computer (MSI MEG Trident X, 12th Gen Intel Core i7, NVIDIA RTX 3080, 32 GB RAM) running Windows 11. Because the timing was critical for this study, participants made button presses using a custom-built grip handle silent button connected to an Arduino micro-controller (<2 ms latency; Schubert et al. 2013) Silent button was used for preventing button press noise to be perceived as the auditory outcome of their actions.

### Temporal recalibration task

**Figure 1:**
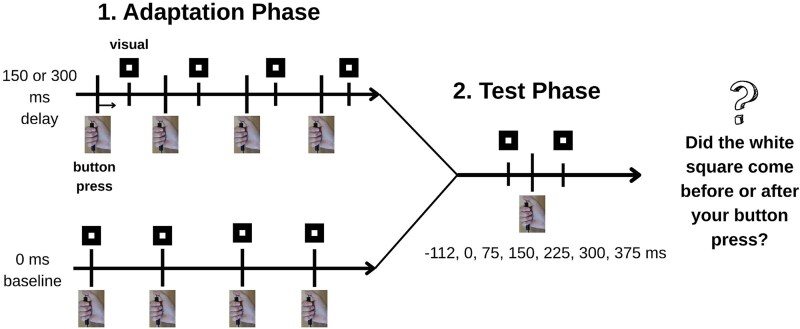
Schematic illustration of the temporal recalibration task. In each trial, participants instructed to perform five consecutive button presses. The first four presses constituted the adaptation phase, during which each press was followed by a visual stimulus (white square) presented at a fixed delay of either 0 ms (baseline condition), 150 ms (short delay condition), or 300 ms (long delay condition). The fifth button press represented the test phase, during which the visual stimulus was presented at one of seven randomly selected stimulus onset asynchronies (SOAs: −112 ms (visual stimulus presented before button press), 0, 75, 150, 225, 300, or 375 ms). Participants made a temporal order judgement, deciding whether the visual stimulus appeared before or after their button press. Conditions were blocked and their order was counterbalanced.

Temporal recalibration task involved adaptation and test phase (Fig. 1) (Stetson et al. 2006; Heron et al. 2009; Sugano et al. 2010). Participants were instructed to make five consecutive button presses with approximately 1 s interval in each trial. The first four button presses constituted the adaptation period, and each button press led to the presentation of a white square with 0 ms (baseline condition), 150 ms (short delay condition), or 300 ms (long delay condition) delay depending on the condition. Timing in the 0 ms baseline condition was checked using high-speed video recording (Sony FDR-AX43A, 100 fps). The button press and white square onset were observed within the same video frame, indicating that any unavoidable system latency was smaller than the temporal resolution of the recording (<10 ms). The fifth button press served as the testing period. On this final press, participants were presented randomly with the visual stimulus (white square) at one of the seven SOAs: −112 (on average, physically before the button press), 0, 75, 150, 225, 300, and 375 ms. On the testing period participants performed a temporal order judgement task where they were asked whether white square appeared before or after their button press. Each SOA was presented for 18 trials in each condition resulting in 126 trials in total for a condition. To present the visual stimulus before the button presses on the testing period, we kept a running average of the participants button press intervals from the adaptation period and predicted their fifth button press timing. We subtracted 75 or 150 ms (first two SOAs in the stimulus set) from the predicted timing of the fifth button press. Using this approach, we were able to present on average 112 ms (SD = 42 ms, Min = 48, ms Max = 199 ms) before the participants’ button press in ‘before’ trials. This average was calculated across all conditions and groups to generate a fixed SOA for ‘before’ trials. This approach was used to avoid possible group and condition dependent differences in SOA sampling influencing the fit and to keep the SOA grid identical across participants and conditions during fitting psychometric functions. As a robustness check, we also repeated our primary analyses using each participant’s observed mean negative SOA separately for each condition when fitting psychometric functions (see section Temporal recalibration results). Delay conditions (0, 150, and 300 ms delay duration conditions) were counterbalanced as no delay condition or delay condition first (Timm et al. 2014) and short and long delay conditions were also counterbalanced within themselves as short delay (150 ms) or long delay (300 ms) condition first. This procedure resulted in these four scenarios (No Delay, Short Delay, Long Delay), (No Delay, Long Delay, Short Delay), (Long Delay, Short Delay, No Delay), (Short Delay, Long Delay, No Delay). Participants completed a practice session involving six trials of 375 ms, 0 ms delay SOAs and visual stimulus before button press SoA in the No-delay condition before the main task started.

## Data analysis

### Point of subjective simultaneity

Each participants’ proportions of ‘after’ responses were calculated for each SoA and experimental condition and modelled by fitting psychometric functions in the form of a cumulative Gaussian distribution function (‘norm’ sigmoid in Psignifit) using Python 3.11 and the Psignifit module (Schütt et al. 2016). The model included three free parameters: the mean (μ); the standard deviation (σ), determining the slope of the function; and a symmetric asymptote parameter accounting for lapse rates at both lower and upper bounds. Point of subjective simultaneity (PSS) as the 50% cut-point of all trials (PSS: the temporal difference needed for button press and visual stimulus to be perceived simultaneous) and just noticeable differences (representing the interval where 27% and 73% after responses were given; Sugano et al. 2010) were calculated for each condition. PSS shifts in the direction of the inserted delay (e.g. PSS shift from No-Delay Condition to Delay Conditions) has been considered as temporal recalibration (Stetson et al. 2006; Heron et al. 2009; Sugano et al. 2010). Hence, temporal recalibration effects (TREs) for Short Delay and Long Delay Conditions were calculated by subtracting PSS value in the No Delay Condition (baseline condition) from the PSS values in these delay conditions.

**Figure 2:**
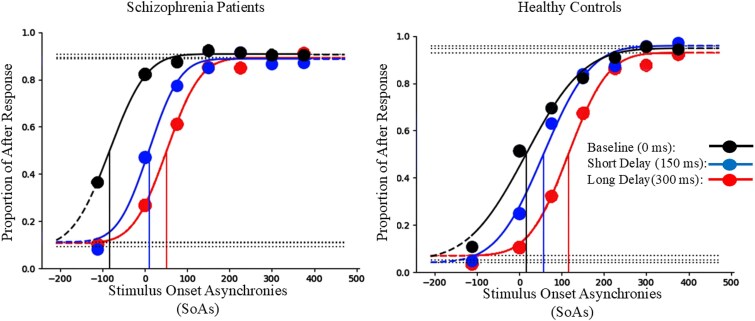
Psychometric functions illustrating temporal order judgements in schizophrenia patients and healthy controls. Group-level proportions of ‘after’ responses were plotted as a function of stimulus onset asynchrony (SOA) and fitted using cumulative Gaussian function. The model estimated the mean (μ; PSS), slope (σ), and a symmetric asymptote parameter accounting for lapse rates. The point of subjective simultaneity (PSS) was defined as the 50% point, where participants were equally likely to judge the visual stimulus as occurring before or after the button press. Functions are presented for the baseline (0 ms delay), short delay (150 ms delay), and long delay (300 ms delay) conditions. Vertical lines indicate the calculated PSS values for each condition, highlighting shifts in perceived simultaneity due to TREs.

For statistical analyses, psychometric functions were fitted separately for each participant and condition, and the resulting individual PSS values were used in all analyses. For visualization purposes (Fig. 2), psychometric functions were additionally estimated at the group level by aggregating responses across participants within each group. Specifically, the total number of ‘after’ responses and trials for each SOA were summed across participants within each group, and the resulting group-level data were then fitted. Thus, the PSS values illustrated in Fig. 2 reflect group-level fitting and may slightly differ from the mean PSS values presented in Table 1.

**Table 1:** Mean (SD) values of point of subjective simultaneity (PSS), just noticeable difference (JND), and temporal recalibration effect (TRE) across conditions for schizophrenia patients and healthy controls

		PSS			JND		TRE	
	Baseline	Short delay	Long delay	Baseline	Short delay	Long delay	Short delay	Long delay
Schizophrenia patients	−67.77 (83.2)	24.17 (64.0)	71.72 (102.9)	88.1 (76.4)	113.1 (95.7)	111 (63.8)	91.94 (59.7)	139.48 (101.7)
Healthy controls	22.47 (94.1)	58.01 (90.2)	124.80 (94.8)	93.5 (63.5)	94.5 (57.8)	94.8 (64.9)	35.5 (63.2)	102.33 (65.0)

## Results

### Temporal recalibration results

**Figure 3:**
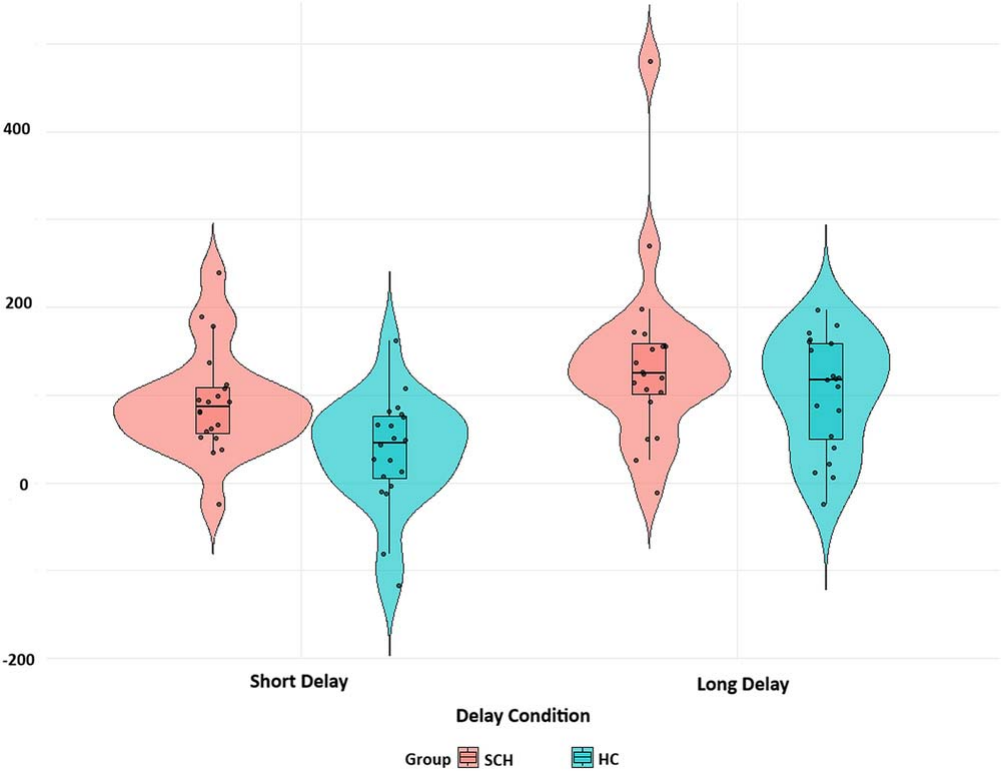
Temporal recalibration effects (TREs) across groups and delay conditions. Individual data points are shown for schizophrenia patients and healthy controls in the short delay and long delay conditions. Boxplots summarize the distribution of TRE values, with the box representing the interquartile range (IQR) and the whiskers indicating the spread of the data within 1.5 × IQR. TRE was calculated by subtracting baseline PSS values from the PSS values observed in each delay condition. Main effects of group (*P* = .028) and condition (*P* < .001) were significant. Group × Condition interaction was not significant (*P* = .408). Visual inspection of the figure indicated the presence of extreme values. Because the design included repeated-measures variable, excluding the entire participant on the basis of a single condition-specific value would have removed valid observations from the remaining condition(s). To ensure that the main ANOVA results were not unduly influenced by extreme observations, we conducted an additional robust 2 × 2 mixed ANOVA using 5% trimmed means. The results of the robust analysis were consistent with those reported in the main results section: The main effects of group, *F*(1, 33.40) = 4.59, *P* = .040, and delay, F(1, 29.38) = 31.25, *P* < .001, remained significant, whereas the Group × Delay interaction remained non-significant, *F*(1, 29.38) = 2.25, *P* = .144. These findings indicate that the main pattern of results was not driven by the extreme values.

To examine group differences in temporal recalibration, we analysed PSS shifts from the No-Delay (Baseline) condition to the two delay conditions (Short and Long Delay conditions), which served as a direct index of TRE. A mixed-design ANOVA was conducted on TREs with group (Schizophrenia Patients and Healthy Controls) as a between-subject variable and condition (Short and Long Delay) as a within-subject variable. This analysis revealed a significant main effect of group [*F* (1,38) = 5.187, *P* = .028, *η^2^_p_* = 0.120] indicating that schizophrenia patients showed significantly greater levels of TREs compared to healthy controls (Fig. 3). As seen in the Table 1, group differences were descriptively more pronounced in the short delay condition where schizophrenia patients had more than two times higher TREs compared to healthy controls on average. We also observed a significant main effect of delay condition, [*F* (1, 38) = 24.71, *P* < .001, η^2^_p_ = 0.394], reflecting generally greater recalibration in the Long Delay condition compared to the Short Delay condition across participants. However, the interaction between group and delay condition was not significant, [*F* (1, 38) = 0.70, *P* = .408], suggesting that the group difference was similar across conditions and the increase in recalibration with longer delays occurred similarly in both groups. Although the group difference appeared larger in the Short Delay condition descriptively, the non-significant interaction indicates that the schizophrenia group showed similarly elevated temporal recalibration across both delay conditions relative to controls.

In the primary temporal recalibration analysis, to maintain an identical SOA grid across groups during psychometric fitting, SOAs in the ‘before’ trials were represented by a fixed grand average (−112 ms) across participants and conditions (see section Temporal recalibration task). To ensure that this averaging procedure did not influence observed group difference in temporal recalibration, we repeated all psychometric fits using each participant’s before SOA separately for each condition and repeated the same analysis above. This analysis showed virtually identical results. The main effect of group, [*F* (1,38) = 5.204, *P* = .028, *η^2^_p_* = 0.120] and delay condition [*F* (1, 38) = 23.855, *P* < .001, η^2^_p_ = 0.386] remained significant and the group × delay condition interaction [*F* (1, 38) = 0.616, *P* = .437] remained non-significant. The changes in the test statistics relative to the primary analysis were negligible, confirming that the larger TRE observed in schizophrenia patients cannot be attributed to the SOA averaging procedure.

### PSS shift and subjective temporal order reversal results

As a control analysis, we investigated whether TRE was observed in both groups. A significant shift of PSS from baseline condition to delay conditions in the direction of the delay would indicate a significant TRE. Schizophrenia patients’ PSS significantly shifted from baseline to short delay, *t* (19) = 6.885, *P* < .001, and to long delay condition (Table 1), *t* (19) = 6.132, *P* < .001. Similarly, healthy controls’ PSS significantly shifted from baseline to short delay, *t* (19) = 2.513, *P* = .021, and to long delay condition (Table 1), *t* (19) = 7.016, *P* < .001. These results suggested that we observed a significant TRE in both groups and conditions. As also suggested by our previous analysis above, schizophrenia patients shifted their PSS more (greater TRE) than healthy controls (Table 1).

In addition to this PSS analysis, we also investigated whether schizophrenia patients experienced more subjective temporal order reversals than healthy controls following the approach described by Stetson et al. (2006). Because the internal reference point (e.g. motor initiation, finger contact, or full key press) participants use to judge the timing of the stimulus is unknown, baseline PSS provides the best available estimate of subjective simultaneity for each participant (Stetson et al. 2006). Accordingly, we quantified subjective temporal order reversals as trials in which the stimulus occurred after the participant’s baseline PSS but was nevertheless reported as occurring before the action in the delay conditions (Stetson et al. 2006). Using this definition, we found that subjective reversals occurred more frequently in schizophrenia patients than in matched healthy controls in the short-delay condition, *t* (38) = 2.427, *P* = .02, whereas the groups did not significantly differ in the long-delay condition, *t* (38) = 0.910, *P* = .368.

### Just noticeable difference results

As another control analysis, a mixed model ANOVA was conducted on the just noticeable difference values (JND) with condition (baseline, short and long delay condition) as within subject and group (schizophrenia patients and healthy controls) as between subject variables. This analysis showed that there was no significant main effect of condition [*F* (2,37) = 0.82, *P* = .447] and group [*F* (1,38) = 0.28, *P* = .600]. The condition × group interaction effect was also not significant [*F* (2,37) = 0.67, *P* = .516]. To further examine whether delay adaptation differentially affected temporal precision across groups, we additionally analysed JND difference scores (Delay condition JND minus baseline condition JND) with condition (short and long delay condition) as within-subject and group (schizophrenia patients and healthy controls) as between-subject variables. There was no significant main effect of condition [*F* (1,38) = 0.006, *P* = .94] and group [*F* (1,38) = 1.35, *P* = .25, η^2^_p_ = 0.034]. Furthermore, the condition x group interaction effect was also not significant [*F* (1,38) = 0.011, *P* = .92]. These results suggest that delay adaptation did not differentially affect temporal precision across groups under the present task conditions.

### Correlations

#### Symptom measures and temporal recalibration

There was no significant correlation between the TRE measures and either SAPS or SANS scores in the schizophrenia group (all |r|s < .25, *P*s > .25, *N* = 20), suggesting that temporal recalibration is not directly associated with symptom severity in this clinically stable sample. This null finding may reflect restricted variance in symptom severity, as participants were outpatients with relatively low levels of symptoms, especially with low levels of positive symptoms. Alternatively, temporal recalibration may reflect a more trait-like, compensatory process that operates independently of current symptom levels. Future research should examine this relationship in larger samples with greater variability in clinical status and symptom severity.

#### Baseline PSS and temporal recalibration

Temporal recalibration is typically defined as the shift in PSS from baseline to delay conditions. Nevertheless, we observed baseline PSS differences between groups: schizophrenia patients exhibited more negative PSS values, *t* (38) = 3.21, *P* = .003, indicating that the visual stimulus had to be presented earlier to be perceived as simultaneous with the action (Table 1). This finding aligns with prior research suggesting slowed and impaired visual processing in schizophrenia (Johnson et al. 2005; Silverstein et al. 2015; Adámek et al. 2022). To further explore the relationship between baseline PSS and temporal recalibration (i.e. the shift in PSS following exposure to a delay), we conducted an exploratory correlation analysis. In the schizophrenia group, baseline PSS was significantly negatively correlated with temporal recalibration in the short delay condition (*r* = −0.64, *P* = .002, *N* = 20), and showed a weaker, non-significant correlation in the long delay condition (*r* = −0.39, *P* = .085, *N* = 20). Healthy controls showed a similar, though non-significant, pattern: baseline PSS correlated negatively with temporal recalibration in both the short delay (*r* = −0.39, *P* = .081) and long delay (*r* = −0.34, *P* = .143) conditions. These findings suggest that greater temporal recalibration in schizophrenia may be partly driven by more negative baseline PSS values, especially under shorter delays.

#### Average daily sleep and temporal recalibration

Interestingly, in an exploratory analysis, we observed that as the patients’ average daily sleep decreased their temporal recalibration scores increased since there was a significant large negative correlation between temporal recalibration in long delay condition and average daily sleep (*r* = −0. 65, *P* = .002, *N* = 20). This might suggest reduced sleep level is associated with more reliance on the compensatory process. There was a similar relationship between the temporal recalibration in the short delay condition and average daily sleep, but it did not reach significance (*r* = −0.35, *P* = .13, *N* = 20). However, in the healthy controls, there was not a significant relationship between temporal recalibration and average daily sleep in the short (*r* = −0.28, *P* = .22, *N* = 20) and long delay condition (*r* = 0.39, *P* = .084, *N* = 20). Given the exploratory and correlational nature of these analyses and the modest sample size, these findings should be interpreted cautiously.

## Discussion

Schizophrenia involves impaired communication and disrupted temporal coordination across widespread brain networks (Uhlhaas et al. 2010; Whitford et al. 2012; Friston et al. 2016; Parlikar et al. 2019). Despite these disturbances, patients often maintain coherent perceptual experiences that support adaptive behaviour, particularly during periods of remission. We propose a dual-role hypothesis of temporal recalibration, a process that compensates for temporal discrepancies in sensorimotor integration. Temporal recalibration may support perceptual coherence during stable phases by mitigating neural incoherence, while also potentially contributing to symptom emergence during periods of temporal instability. This hypothesis complements neural network dysconnectivity theories by providing a specific temporal mechanism that could account for the shifts between functional and fragmented perception in schizophrenia. Specifically, we focused on its compensatory role and tested whether clinically stable patients exhibit greater sensorimotor temporal recalibration than healthy controls.

### Enhanced visuomotor temporal recalibration in schizophrenia

Our results provide empirical evidence that schizophrenia patients demonstrate greater degrees of sensorimotor temporal recalibration in the visuomotor domain. This finding suggests an increased reliance on temporal realignment process to persistent sensorimotor temporal asynchronies. This emphasizes the possible compensatory role of sensorimotor temporal recalibration in counteracting neural network dysconnectivity and facilitating functional perception and effective interaction with the environment in clinically stable patients. Sensorimotor temporal asynchronies requiring increased levels of reliance on compensatory temporal adjustment process might stem from known abnormalities in the timing and integration of wide-spread neural activity. These include impaired efference copy (Whitford et al. 2012; Pynn and DeSouza 2013; Parlikar et al. 2019), slowed or less efficient visual processing (Johnson et al. 2005; Silverstein et al. 2015; Jiang et al. 2021; Adámek et al. 2022; Francisco et al. 2022), reduced connectivity in fronto-occipital network (Khadka et al. 2013) and cerebellum related dysfunction (Andreasen and Pierson 2008; Kim et al. 2021). These abnormalities may lead to asynchronies across sensorimotor systems that needs to be temporally coordinated by overuse of temporal recalibration. Our finding suggests that schizophrenia patients not only have access to the temporal recalibration mechanism but also may recruit it more than healthy individuals to mitigate disconnection-related delays and preserve temporal coherence. Therefore, temporal recalibration may serve as a temporal correction strategy in schizophrenia during remission.

### Predictive processing account of exaggerated temporal recalibration

Our findings can also be interpreted within the predictive processing and Bayesian inference frameworks of schizophrenia. According to this view, increased precision weighting of sensory evidence leads to exaggerated updating of relatively low-precision internal predictions; a biased precision-weighting process thought to be central to perceptual and cognitive disturbances in schizophrenia (Fletcher and Frith 2009; Adams et al. 2013; Clark 2013; Friston et al. 2016; Sterzer et al. 2018). Temporal recalibration can be conceptualized as a process by which the brain dynamically adjusts its timing expectations in response to repeated exposure to temporal asynchronies (Stetson et al. 2006; Parsons et al. 2013). Bayesian views of temporal recalibration suggest that this dynamic process involves updating internal priors to minimize prediction errors between expected and actual temporal relationships (Di Luca et al. 2009; Sadibolova and Terhune 2022). The robust temporal recalibration observed in schizophrenia may reflect the above-described biased precision-weighting. That is, unreliable internal timing predictions are overridden by high-precision incoming evidence, resulting in exaggerated updating of temporal expectations. This is reflected in greater shifts in the PSS based on prior experience and consequently in greater levels of temporal recalibration. This interpretation is also compatible with developmental findings suggesting reduced recalibration observed in children may arise from low-precision sensory representations, making incoming temporal evidence less effective for updating prior expectations (Vercillo et al. 2015; Shi and Burr 2016). Together, these patterns of findings suggest different sources of uncertainty may result in different levels of temporal recalibration.

### Temporal recalibration and temporal precision

In the present study, schizophrenia patients had statistically similar temporal precision to their matched healthy controls, both in the JND values or in JND difference scores relative to baseline. This differs from studies reporting broader temporal windows in schizophrenia (e.g. Martin et al. 2013; Haß et al. 2017; Thoenes and Oberfeld 2017; Zvyagintsev et al. 2017). One possible reason for this discrepancy is methodological: prior studies primarily examined audiovisual temporal integration, whereas the present study assessed sensorimotor temporal recalibration. In addition, the relatively large SOAs used here to ensure that the task was feasible for both groups may have reduced sensitivity to subtle group differences in temporal precision. The absence of group differences in JND suggests that the enhanced temporal recalibration observed in schizophrenia patients cannot be readily explained by a general deficit in temporal sensitivity or overall task difficulty in the present study. Instead, the findings are more consistent with the idea that schizophrenia patients relied more heavily on recalibration mechanisms in response to delayed sensory feedback. In this sense, the greater temporal recalibration in schizophrenia appears to reflect altered adaptation of subjective simultaneity rather than a widening of temporal uncertainty.

### Potential maladaptive consequences

Although increased sensorimotor temporal recalibration may serve an adaptive role, it may also have disruptive consequences. Temporal recalibration studies in healthy individuals showed that adaptation to action-outcome temporal asynchronies (e.g. 150 ms delay) can reverse subjective temporal order, especially when a stimulus is presented transiently earlier than the adapted delay (Stetson et al. 2006; Heron et al. 2009; Sugano et al. 2010). In the present study, schizophrenia patients showed larger shifts in the PSS from baseline to delay conditions than healthy controls. This indicates that, in the patient group, stimuli that were previously reported as occurring after the action in the baseline condition were more likely to be reported as occurring before the action following delay adaptation. Additionally, following the approach described by Stetson et al. (2006), we quantified subjective temporal order reversals as trials in which the stimulus occurred after the participant’s baseline PSS but was reported as occurring before the action. We found that these subjective reversals occurred more frequently in schizophrenia patients than in matched healthy controls in the short delay (150 ms) condition, whereas the groups did not significantly differ in the long delay (300 ms) condition. These findings are consistent with the larger PSS shifts observed in the patient group and suggest that schizophrenia patients may be more susceptible to recalibration-induced subjective reversals of temporal order under certain delay conditions.

Such temporal reversals may contribute to disruptions in the sense of agency (Timm et al. 2014), the experience of control over our actions and their consequences. It is possible that dysconnectivity-related temporal asynchronies across neural systems lead schizophrenia patients to rely more heavily on recalibration processes in order to maintain temporal coherence. However, increased levels of temporal recalibration can also increase the risk of experiencing temporal order reversals, especially when transient changes in the adapted asynchronies occur. During acute phases, neural responsivity and excitability can be increased transiently by heightened arousal and neurochemical changes, particularly dopaminergic dysregulation (Lodge and Grace 2011; Grace 2012; McCutcheon et al. 2020; Corlett and Fraser 2025) and may transiently alter previously recalibrated timing relationships across neural systems. Consequently, processing speeds may temporarily increase and reduce the adapted temporal asynchronies and reverse perceived temporal order; thereby disrupting causality and the sense of agency. This instability may contribute to the emergence of positive symptoms such as diminished agency, thought insertion, and hallucinations by altering the perceived timing of internally generated experiences. Moreover, temporal recalibration is known to transfer across sensory modalities (Heron et al. 2009; Sugano et al. 2010), and such cross-modal transfer from visual to auditory domains could distort the temporal structure of internal speech. If internal speech or thoughts are perceived as occurring before their initiating intention, they may be experienced as alien or externally inserted. This suggests that exaggerated temporal recalibration in visual domain may also have disruptive effects on auditory domain. Therefore, we suggest that, especially when transient changes in processing speed occur, overreliance on temporal recalibration mechanism has the potential to contribute to positive symptoms and this disruptive effect may generalize to other modalities.

### Modality-specific effects and task-related considerations

A recent study in schizophrenia patients found that their motor-auditory temporal recalibration was not significantly different than healthy controls (Schmitter and Straube 2024). This contrasts with our findings in the visual modality, where we observed clear group differences. Differential temporal sensitivity across sensory modalities may help explain this discrepancy. Auditory system is known for its superior temporal resolution and faster information processing (Kanai et al. 2011) which might require less recalibration to maintain smaller asynchronies or may be less affected by internal delays (Di Luca et al. 2009; Aytemür et al. 2017). On the contrary, visual system is slower and more prone to schizophrenia-related delays (Johnson et al. 2005; Silverstein et al. 2015; Jiang et al. 2021; Adámek et al. 2022; Francisco et al. 2022) which can potentially put greater demand for temporal recalibration. It has been also suggested that visual temporal processing differences across the schizophrenia spectrum may involve dysregulated temporal sampling, or an altered balance between temporal segregation and integration (Deodato et al. 2024). Based on these, heightened motor-visual temporal recalibration may reflect a compensatory response to disrupted visual timing, helping maintain action-outcome synchrony when baseline sensorimotor timing is unstable.

Methodological differences across studies may also contribute to the discrepancy. Schmitter and Straube (2024) employed a simultaneity judgement task in which the auditory stimulus coincided with or followed the action, whereas our paradigm included SOAs that could occur physically before the button press by predicting the upcoming press from preceding inter-press intervals. This SOA range allowed us to detect negative PSS in the baseline condition of schizophrenia patients (Fig. 2). That is, visual stimulus needed to occur earlier to be perceived as simultaneous with the action and this finding is consistent with the visual processing lags in schizophrenia. Also, our exploratory correlational findings between baseline PSS and temporal recalibration suggest that greater temporal recalibration in schizophrenia may be driven by more negative baseline PSS values, especially under shorter delays. This supports the idea that increased temporal recalibration may serve a compensatory function when baseline sensorimotor synchrony is disrupted. It also suggests that the inclusion of negative SOAs is useful to capture the extend of temporal recalibration in schizophrenia. Future studies investigating both visual and auditory sensorimotor temporal recalibration is needed to uncover the modality specific effects in schizophrenia.

### Exploratory sleep findings, limitations, and future directions

Our exploratory correlation analysis showed that shorter self-reported average sleep duration was associated with larger temporal recalibration in patients, particularly in the long-delay condition. One possible interpretation is that shorter sleep durations may be associated with increased reliance on compensatory mechanisms for resolving sensorimotor timing discrepancies in schizophrenia. This might be due to the potential effects of shorter sleep duration on already fragile predictive or integrative processes. Considering previous findings linking sleep disruption to impaired cognitive function and synaptic coordination (Durmer and Dinges 2005; Tononi and Cirelli 2014), the correlations we observed offer preliminary support for the view that temporal recalibration may act as an adaptive mechanism when cognitive resources and neural coordination are compromised. However, it should also be considered that shorter sleep duration does not necessarily reflect sleep deprivation; some individuals may be habitual short sleepers who report adequate functioning, and we did not assess sleep quality, daytime sleepiness, or objective sleep parameters. Also, exploratory and correlational nature of this finding in a relatively small sample warrant caution. Future studies should investigate the causal dynamics between objective sleep parameters, temporal recalibration, and predictive function.

There are limitations to this study that should be acknowledged. First, a methodological limitation concerns the range of SOAs used in the testing phase. In the baseline condition psychometric functions in the schizophrenia group did not reach the lower asymptote whereas this was not the case in healthy control group. This suggests earliest SOA (−112 ms on average) may not have been sufficiently negative to fully capture the PSS in schizophrenia. Consequently, this limitation likely resulted in a less negative baseline PSS than the true value which in turn implies that our estimation of temporal recalibration may be conservative in schizophrenia since temporal recalibration was calculated as the PSS shift from baseline. Therefore, future studies employing wider SOA ranges, particularly with more negative values, may capture more accurate estimation of temporal recalibration in patients. Second, although our sample size is comparable to other studies in the area (Johnson et al. 2005; Stetson et al. 2006; Schmitter and Straube 2024), it is modest. Importantly, however, we observed a statistically significant group difference in temporal recalibration, with a moderate-to-large effect size (ηp^2^ = 0.120). This suggests that the observed effect is both robust and theoretically meaningful. Nevertheless, further studies with larger and more diverse samples are warranted to replicate and extend these findings, and to explore potential moderators such as medication status, symptom severity, or cognitive functioning. Third, in the current study we specifically focused on the visual modality based on previous findings suggesting slower and delayed visual functioning in schizophrenia. However, whether temporal recalibration profiles would be similar in other sensory domains and multisensory contexts needs to be examined. Finally, although neural connectivity problems have been widely shown in the literature and we propose a compensatory role for temporal recalibration in mitigating these, causality cannot be inferred from the present data. This might be tackled with using both neuroimaging and brain stimulation techniques in a similar procedure in the future studies.

## Conclusion

In conclusion, our hypothesis suggests that sensorimotor temporal recalibration may play a previously underrecognized role in schizophrenia, serving both as a compensatory mechanism during remission and a potential contributor to symptom emergence under conditions of temporal instability. We provide evidence that clinically stable patients exhibit exaggerated visuomotor temporal recalibration indicating that the mechanism remains functionally accessible and may be over-engaged to mitigate neural asynchronies. At the same time, we propose that the same mechanism may become maladaptive. When already adapted asynchronies are disrupted, potentially because of transient changes in information processing speed, the temporal recalibration mechanism may distort the perceived order of actions and outcomes. These distortions may contribute to positive symptoms such as hallucinations and delusions of control, particularly by disrupting sense of agency.

This dual-role hypothesis attempts to bridge adaptive perceptual timing, predictive processing, and clinical symptomology, and offers a novel perspective on how schizophrenia patients may cope with or be destabilized by neural dysconnectivity. Future studies incorporating neural measures, neuromodulation, and symptom specific subgroup comparisons may be essential for testing the boundaries and dynamics of this temporal compensation mechanism. Notably, sensorimotor temporal recalibration has been shown to be modifiable by non-invasive brain stimulation such as transcranial direct current stimulation in healthy individuals (Aytemür et al. 2017; Schmitter and Straube 2022), highlighting its potential for safe, accessible, and targeted modulation. In addition, transcranial alternating current stimulation has been shown to modulate temporal integration or sampling windows in both multisensory and unisensory contexts (Cecere et al., 2015; Santoni et al., 2025), suggesting that it may be especially well suited for targeting oscillatory mechanisms underlying temporal coordination. Taken together, these insights may provide a basis for both a mechanistic framework for the temporal organization of perception in schizophrenia and open new avenues for translational research.

## Supplementary Material

Supplementary_materials_niag031

## Data Availability

The data that support the findings of this study are available from the corresponding author, A.A., upon reasonable request.

## References

[ref1] Adámek P, Langová V, Horáček J. Early-stage visual perception impairment in schizophrenia, bottom-up and back again. *Schizophrenia* 2022;8:27. 10.1038/s41537-022-00237-9 35314712 PMC8938488

[ref2] Adams RA, Stephan KE, Brown HR et al. The computational anatomy of psychosis. *Front Psychiatry* 2013;4:47. 10.3389/fpsyt.2013.00047 23750138 PMC3667557

[ref3] Andreasen NC . The Scale for the Assessment of Positive Symptoms (SAPS). Iowa City, IA: University of Iowa, 1984a.

[ref4] Andreasen NC . The Scale for the Assessment of Negative Symptoms (SANS). Iowa City, IA: University of Iowa, 1984b.

[ref5] Andreasen NC, Pierson R. The role of the cerebellum in schizophrenia. *Biol Psychiatry* 2008;64:81–8. 10.1016/j.biopsych.2008.01.003 18395701 PMC3175494

[ref6] Andreou C, Faber PL, Leicht G et al. Resting-state connectivity in the prodromal phase of schizophrenia: insights from EEG microstates. *Schizophr Res* 2014;152:513–20. 10.1016/j.schres.2013.12.008 24389056

[ref7] Assaf R, Ouellet J, Bourque J et al. Resting-state alterations in emotion salience and default-mode network connectivity in atypical trajectories of psychotic-like experiences. *Dev Psychopathol* 2024;37:1495–504. 10.1017/S0954579424001317 39297232

[ref8] Aytemür A, Almeida N, Lee KH. Differential sensory cortical involvement in auditory and visual sensorimotor temporal recalibration: evidence from transcranial direct current stimulation (tDCS). *Neuropsychologia* 2017;96:122–8. 10.1016/j.neuropsychologia.2017.01.012 28089696

[ref1c] Cecere R, Rees G, Romei V. Individual differences in alpha frequency drive crossmodal illusory perception. Current Biology, 2015;25:231–235. 25544613 10.1016/j.cub.2014.11.034 PMC4300399

[ref9] Clark A . Whatever next? Predictive brains, situated agents, and the future of cognitive science. *Behav Brain Sci* 2013;36:181–204. 10.1017/S0140525X12000477 23663408

[ref10] Corlett PR, Fraser KM. 20 years of aberrant salience in psychosis: what have we learned? *Am J Psychiatry* 2025;182:819–29. 10.1176/appi.ajp.20240556 40134268 PMC12949980

[ref11] Del Fabro L, Schmidt A, Fortea L et al. Functional brain network dysfunctions in subjects at high-risk for psychosis: a meta-analysis of resting-state functional connectivity. *Neurosci Biobehav Rev* 2021;128:90–101. 10.1016/j.neubiorev.2021.06.020 34119524

[ref12] Deodato M, Ronconi L, Melcher D. Schizotypal traits and anomalous perceptual experiences are associated with greater visual temporal acuity. *Schizophr Res* 2024;269:1–8. 10.1016/j.schres.2024.04.028 38703518

[ref13] Di Luca M, Machulla TK, Ernst MO. Recalibration of multisensory simultaneity: cross-modal transfer coincides with a change in perceptual latency. *J Vis* 2009;9:7.1–16. 10.1167/9.12.7 20053098

[ref14] Durmer JS, Dinges DF. Neurocognitive consequences of sleep deprivation. *Semin Neurol* 2005;25:117–29. 10.1055/s-2005-867080 15798944

[ref15] Erkoç Ş, Arkonaç O, Ataklı C et al. Pozitif semptomları değerlendirme ölçeğinin güvenilirliği ve geçerliliği. *Düşünen Adam* 1991a;4:20–4.

[ref16] Erkoç Ş, Arkonaç O, Ataklı C et al. Negatif semptomları değerlendirme ölçeğinin güvenilirliği ve geçerliliği. *Düşünen Adam* 1991b;4:14–5.

[ref17] First MB . Structured Clinical Interview for DSM-IV Axis I Disorders (SCID-I), Clinician Version (Administration Booklet). Washington, DC: American Psychiatric Publishing, Inc., 1997.

[ref18] Fletcher PC, Frith CD. Perceiving is believing: a Bayesian approach to explaining the positive symptoms of schizophrenia. *Nat Rev Neurosci* 2009;10:48–58. 10.1038/nrn2536 19050712

[ref19] Ford JM, Mathalon DH. Neural synchrony in schizophrenia. *Schizophr Bull* 2008;34:904–6. 10.1093/schbul/sbn090 18658126 PMC2632484

[ref20] Francisco AA, Foxe JJ, Horsthuis DJ et al. Early visual processing and adaptation as markers of disease, not vulnerability: EEG evidence from 22q11.2 deletion syndrome, a population at high risk for schizophrenia. *Schizophrenia* 2022;8:28. 10.1038/s41537-022-00240-0 35314711 PMC8938446

[ref21] Friston K, Brown HR, Siemerkus J et al. The dysconnection hypothesis. *Schizophr Res* 2016;176:83–94. 10.1016/j.schres.2016.07.014 27450778 PMC5147460

[ref22] Fujisaki W, Shimojo S, Kashino M et al. Recalibration of audiovisual simultaneity. *Nat Neurosci* 2004;7:773–8. 10.1038/nn1268 15195098

[ref23] Grace AA . Dopamine system dysregulation by the hippocampus: implications for the pathophysiology and treatment of schizophrenia. *Neuropharmacology* 2012;62:1342–8. 10.1016/j.neuropharm.2011.05.011 21621548 PMC3179528

[ref24] Haß K, Sinke C, Reese T et al. Enlarged temporal integration window in schizophrenia indicated by the double-flash illusion. *Cogn Neuropsychiatry* 2017;22:145–58. 10.1080/13546805.2017.1287693 28253091

[ref25] Heron J, Hanson JV, Whitaker D. Effect before cause: supramodal recalibration of sensorimotor timing. *PLoS One* 2009;4:e7681. 10.1371/journal.pone.0007681. 19890383 PMC2766625

[ref26] Jiang CG, Wang J, Liu XH et al. The neural correlates of effortful cognitive processing deficits in schizophrenia: an ERP study. *Front Hum Neurosci* 2021;15:664008. 10.3389/fnhum.2021.664008 34122029 PMC8193231

[ref27] Johnson SC, Lowery N, Kohler C et al. Global–local visual processing in schizophrenia: evidence for an early visual processing deficit. *Biol Psychiatry* 2005;58:937–46. 10.1016/j.biopsych.2005.04.053 16084856

[ref28] Kanai R, Lloyd H, Bueti D et al. Modality-independent role of the primary auditory cortex in time estimation. *Exp Brain Res* 2011;209:465–71. 10.1007/s00221-011-2577-3 21318347

[ref29] Khadka S, Meda SA, Stevens MC et al. Is aberrant functional connectivity a psychosis endophenotype? A resting state functional magnetic resonance imaging study. *Biol Psychiatry* 2013;74:458–66. 10.1016/j.biopsych.2013.04.024 23746539 PMC3752322

[ref30] Kim SE, Jung S, Sung G et al. Impaired cerebro-cerebellar white matter connectivity and its associations with cognitive function in patients with schizophrenia. *NPJ Schizophr* 2021;7:38. 10.1038/s41537-021-00169-w 34385473 PMC8360938

[ref31] Lodge DJ, Grace AA. Hippocampal dysregulation of dopamine system function and the pathophysiology of schizophrenia. *Trends Pharmacol Sci* 2011;32:507–13. 10.1016/j.tips.2011.05.001 21700346 PMC3159688

[ref32] Martin B, Giersch A, Huron C et al. Temporal event structure and timing in schizophrenia: preserved binding in a longer “now”. *Neuropsychologia* 2013;51:358–71. 10.1016/j.neuropsychologia.2012.07.002 22813430

[ref33] Mathôt S, Schreij D, Theeuwes J. OpenSesame: an open-source, graphical experiment builder for the social sciences. *Behav Res Methods* 2012;44:314–24. 10.3758/s13428-011-0168-7 22083660 PMC3356517

[ref34] McCutcheon RA, Krystal JH, Howes OD. Dopamine and glutamate in schizophrenia: biology, symptoms and treatment. *World Psychiatry* 2020;19:15–33. 10.1002/wps.20693 31922684 PMC6953551

[ref35] Parlikar TA, Cahill CM, Whitford TJ. Altered myelination as a contributor to schizophrenia: evidence from diffusion tensor imaging studies. *Front Psychiatry* 2019;10:173.30984045

[ref36] Parsons BD, Novich SD, Eagleman DM. Motor-sensory recalibration modulates perceived simultaneity of cross-modal events at different distances. *Front Psychol* 2013;4:46. 10.3389/fpsyg.2013.00046 23549660 PMC3582016

[ref37] Pelletier-Baldelli A, Andrews-Hanna JR, Mittal VA. Resting state connectivity dynamics in individuals at risk for psychosis. *J Abnorm Psychol* 2018;127:314–25. 10.1037/abn0000330 29672091 PMC5912697

[ref38] Phillips WA, Silverstein SM. Convergence of biological and psychological perspectives on cognitive coordination in schizophrenia. *Behav Brain Sci* 2003;26:65–82. 10.1017/S0140525X03000025 14598440

[ref39] Pynn LK, DeSouza JF. The function of efference copy signals: implications for symptoms of schizophrenia. *Vis Res* 2013;76:124–33. 10.1016/j.visres.2012.10.019 23159418

[ref40] Sadibolova R, Terhune DB. The temporal context in Bayesian models of interval timing: recent advances and future directions. *Behav Neurosci* 2022;136:364–73. 10.1037/bne0000513 35737557 PMC9552499

[ref1s] Santoni A, Di Dona G, Gironi R et al. Bifocal alpha-band tACS modulates temporal sampling in visual perception. NeuroImage, 2025;121474. 10.1016/j.neuroimage.2025.121474 40976493

[ref41] Schmitter CV, Straube B. The impact of cerebellar transcranial direct current stimulation (tDCS) on sensorimotor and inter-sensory temporal recalibration. *Front Hum Neurosci* 2022;16:998843. 10.3389/fnhum.2022.998843 36111210 PMC9468227

[ref42] Schmitter CV, Straube B. Facilitation of sensorimotor temporal recalibration mechanisms by cerebellar tDCS in patients with schizophrenia spectrum disorders and healthy individuals. *Sci Rep* 2024;14:2627. 10.1038/s41598-024-53148-3 38297015 PMC10830570

[ref43] Schubert TW, D’Ausilio A, Canto R. Using Arduino microcontroller boards to measure response latencies. *Behav Res Methods* 2013;45:1332–46. 10.3758/s13428-013-0336-z 23585023

[ref44] Schütt HH, Harmeling S, Macke JH et al. Painfree and accurate Bayesian estimation of psychometric functions for (potentially) overdispersed data. *Vis Res* 2016;122:105–23. 10.1016/j.visres.2016.02.002 27013261

[ref2s] Shi Z, Burr D. Predictive coding of multisensory timing. Current Opinion in Behavioral Sciences, 2016;8:200–206. 27695705 10.1016/j.cobeha.2016.02.014 PMC5040498

[ref45] Silverstein S, Keane BP, Blake R et al. Vision in schizophrenia: why it matters. *Front Psychol* 2015;6:41. 10.3389/fpsyg.2015.00041 25698992 PMC4318337

[ref46] Sterzer P, Adams RA, Fletcher P et al. The predictive coding account of psychosis. *Biol Psychiatry* 2018;84:634–43. 10.1016/j.biopsych.2018.05.015 30007575 PMC6169400

[ref47] Stetson C, Cui X, Montague PR et al. Motor-sensory recalibration leads to an illusory reversal of action and sensation. *Neuron* 2006;51:651–9. 10.1016/j.neuron.2006.08.006 16950162

[ref48] Sugano Y, Keetels M, Vroomen J. Adaptation to motor-visual and motor-auditory temporal lags transfer across modalities. *Exp Brain Res* 2010;201:393–9. 10.1007/s00221-009-2047-3 19851760 PMC2832876

[ref49] Thoenes S, Oberfeld D. Meta-analysis of time perception and temporal processing in schizophrenia: differential effects on precision and accuracy. *Clin Psychol Rev* 2017;54:44–64. 10.1016/j.cpr.2017.03.007 28391027

[ref50] Timm J, Schönwiesner M, SanMiguel I et al. Sensation of agency and perception of temporal order. *Conscious Cogn* 2014;23:42–52. 10.1016/j.concog.2013.11.002 24362412

[ref51] Tononi G, Cirelli C. Sleep and the price of plasticity: from synaptic and cellular homeostasis to memory consolidation and integration. *Neuron* 2014;81:12–34. 10.1016/j.neuron.2013.12.025 24411729 PMC3921176

[ref52] Uhlhaas PJ, Singer W. Abnormal neural oscillations and synchrony in schizophrenia. *Nat Rev Neurosci* 2010;11:100–13. 10.1038/nrn2774 20087360

[ref1v] Vercillo T, Burr D, Sandini G, Gori M. Children do not recalibrate motor‐sensory temporal order after exposure to delayed sensory feedback. Developmental science, 2015;18:703–712. 25444457 10.1111/desc.12247 PMC4487828

[ref53] Vroomen J, Keetels M, de Gelder B et al. Recalibration of temporal order perception by exposure to audio-visual asynchrony. *Cogn Brain Res* 2004;22:32–5. 10.1016/j.cogbrainres.2004.07.003 15561498

[ref54] Wang C, Ji F, Hong Z et al. Disrupted salience network functional connectivity and white-matter microstructure in persons at risk for psychosis: findings from the LYRIKS study. *Psychol Med* 2016;46:2771–83. 10.1017/S0033291716001410 27396386 PMC5358474

[ref55] Whitford TJ, Ford JM, Mathalon DH et al. Schizophrenia, myelination, and delayed corollary discharges: a hypothesis. *Schizophr Bull* 2012;38:486–94. 10.1093/schbul/sbq105 20855415 PMC3329979

[ref56] Zvyagintsev M, Parisi C, Mathiak K. Temporal processing deficit leads to impaired multisensory binding in schizophrenia. *Cogn Neuro-psychiatry* 2017;22:361–72. 10.1080/13546805.2017.1331160 28578638

