# Supplementary material for: Temporal recalibration in schizophrenia: a compensatory timing trap?
Source: Neurosci Conscious. 2026 Jun 22;2026(1):niag031. doi: 10.1093/nc/niag031 (PMC13286012; doi:10.1093/nc/niag031)
Supplement: Supplementary_materials_niag031 [file supplementary_materials_niag031.docx]

| Variable | Schizophrenia (n = 20) | Healthy Controls (n = 20) |
| --- | --- | --- |
| Gender (Male %) | 60% | 60% |
| Age, M (SD) | 38.90 (9.79) | 39.60 (9.29) |
| Education (years), M (SD) | 13.00 (3.93) | 11.85 (3.36) |
| Socioeconomic Status, M (SD) | 4.65 (1.57) | 5.75 (1.41) |
| SAPS Total, M (SD) | 6.20 (6.92) | - |
| SANS Total, M (SD) | 15.45 (14.08) | - |
| Hallucinations, M (SD) | 1.25 (2.51) | - |
| Delusions, M (SD) | 1.65 (2.35) | - |
| Bizarre Behavior, M (SD) | 0.80 (1.20) | - |
| Formal Thought Disorder, M (SD) | 2.50 (2.89) | - |
| Affective Flattening, M (SD) | 3.50 (4.97) | - |
| Alogia, M (SD) | 1.80 (2.78) | - |
| Avolition, M (SD) | 2.40 (2.64) | - |
| Anhedonia-Asociality, M (SD) | 5.20 (4.69) | - |
| Attention, M (SD) | 2.55 (2.35) | - |

Supplementary Table 1 Demographic and Clinical Characteristics of the Schizophrenia and Control Groups

Supplementary Table 2 Medication details of patients

| Participant number | Antipsychotic medication(s) | Concomitant medication(s) |
| --- | --- | --- |
| 1 | Clozapine (200 mg), Aripiprazole (15 mg) | Citalopram (20 mg), Propranolol (40 mg) |
| 2 | Zuclopenthixol (200 mg / 2 weeks, depot), Quetiapine (900 mg) | Zopiclone (7.5 mg), Escitalopram (20 mg) |
| 3 | Haloperidol (20 mg), Olanzapine (20 mg), Zuclopenthixol (200 mg / 3 weeks, depot) | — |
| 4 | Clozapine (150 mg) | Amitriptyline (25 mg) |
| 5 | Aripiprazole (400 mg / month, depot) | — |
| 6 | Clozapine (200 mg) | Lamotrigine (100 mg), Sertraline (200 mg) |
| 7 | Paliperidone (6 mg) | — |
| 8 | Clozapine (300 mg), Aripiprazole (30 mg) | Sertraline (100 mg) |
| 9 | Olanzapine (10 mg), Aripiprazole (30 mg) | — |
| 10 | Risperidone (50 mg / 2 weeks, depot), Olanzapine (10 mg) | — |
| 11 | Aripiprazole (30 mg), Clozapine (625 mg) | Sertraline (150 mg) |
| 12 | Paliperidone (3 mg), Aripiprazole (10 mg) | Escitalopram (20 mg), |
| 13 | Paliperidone (50 mg / month, depo), Aripiprazole (5 mg), Quetiapine (100 mg) | Escitalopram (10 mg),  Biperiden (2 mg) |
| 14 | Paliperidone (6 mg) | — |
| 15 | Olanzapine (5 mg) | — |
| 16 | Paliperidone (100 mg / month, depot) | Biperiden (2 mg) |
| 17 | Aripiprazole (5 mg), Clozapine (50 mg), Paliperidone (100 mg / month, depot) | — |
| 18 | Clozapine (175 mg), Aripiprazole (20 mg) | Lithium (300 mg) |
| 19 | Paliperidone (150 mg depot), Clozapine (200 mg) | Venlafaxine (150 mg) |
| 20 | Clozapine (175 mg), Paliperidone (150 mg depot) | Sertraline (100 mg), Valproic acid (1000 mg) |
